# Crystal structure of the uranyl arsenate mineral hügelite, Pb_2_(UO_2_)_3_O_2_(AsO_4_)_2_(H_2_O)_5_, revisited: a correct unit cell, twinning and hydrogen bonding

**DOI:** 10.1107/S2052520621004091

**Published:** 2021-05-14

**Authors:** Jakub Plášil, Václav Petříček, Pavel Škácha

**Affiliations:** a Institute of Physics ASCR, v.v.i., Na Slovance 2, Praha 8, 18221, Czech Republic; b Department of Mineralogy and Petrology, National Museum, Cirkusová 1740, Praha 9, 19300, Czech Republic

**Keywords:** hügelite, uranyl arsenate, crystal structure, twinning, *JANA2006*, reticular merohedry

## Abstract

Revisiting the structure of uranyl arsenate mineral hügelite provided some corrections to the available structural data. The previous twinning model (by reticular merohedry) in hügelite has been corrected. Twinning of the monoclinic unit cell [*a* = 7.0189 (7) Å, *b* = 17.1374 (10) Å, *c* = 8.1310 (10) Å and β = 108.904 (10)°], which can be expressed as a mirror in [100], leads to a pseudo-orthorhombic unit cell (*a* = 7.019 Å, *b* = 17.137 Å, *c* = 61.539 Å and β = 90.02°), which is eight times larger, with respect to the unit-cell volume, than a real cell. Moreover, the unit cell of chosen here and the unit cell given by the previous structure description both lead to the same supercell. A new structure refinement undertaken on an untwinned crystal of hügelite resulted in *R* = 4.82% for 12 864 reflections with *I*_obs_ > 3σ(*I*) and GOF = 1.12. The hydrogen-bonding scheme has been proposed for hügelite for the first time.

## Abstract 2

The crystal structure of the uranyl arsenate mineral hügelite is affected by twinning due to reticular merohedry (diffraction type II). This study documents, apart from the correct description of the unit cell and the nature of the twinning, the possibilities of the *JANA2006* program in revealing the real nature of twinning even if using published structural data without the original reflection files.

## 1. Introduction   

Uranyl arsenates are, along with uranyl phosphates, the most common alteration products after oxidation weathering of uraninite (Finch & Murakami, 1999 ▸; Krivovichev & Plášil, 2013 ▸; Plášil, 2014 ▸). They occur in the uppermost oxidized parts of uranium deposits, surviving the near-surface or surface con­ditions due to their very low solubility in aqueous solutions (*e.g.* Vochten & Goeminne, 1984 ▸; Gorman-Lewis *et al.*, 2009 ▸; Maher *et al.*, 2013 ▸). The most common uranyl arsenates com­prise minerals of the well-known autunite group: a Cu-member zeunerite, Cu[(UO_2_)(AsO_4_)]_2_(H_2_O)_12_, and a Ca-mem­ber uranospinite, Ca[(UO_2_)(AsO_4_)]_2_(H_2_O)_10_. The mineral hügelite, Pb_2_[(UO_2_)_3_O_2_(AsO_4_)_2_](H_2_O)_5_, was described originally as a lead–zinc vanadate hydrate (Dürrfeld, 1913 ▸), but was redetermined later (Walenta & Wimmenauer, 1961 ▸) as a lead uranyl arsenate hydrate, structurally related to dumontite, Pb_2_[(UO_2_)_3_O_2_(PO_4_)_2_]·5H_2_O (Piret & Piret-Meunier, 1988 ▸). Later on, it was investigated again, from the material originating from the type locality, which is the Michael Mine at Weiler, near Lahr in the Black Forest, Baden–Württemberg, Germany (Walenta, 1979 ▸). This study updated morphological crystallographic data and physical and optical properties, providing a new formula, Pb_2_(UO_2_)_3_(AsO_4_)_2_(OH)_4_·3H_2_O, and produced new crystallographic data. According to that study, hügelite is monoclinic, space group *P*2_1_/*m* or *P*2_1_, with *a* = 8.13 Å, *b* = 17.27 Å, *c* = 7.01 Å, β = 109°, *Z* = 2 and *D*_calc_ = 5.80 Mg m^−3^. In 2003, Locock & Burns (2003 ▸) undertook a modern crystallographic study based on a crystal originating from ‘Geroldseck, Baden’, Germany. Based on the diffraction experiment using a four-circle diffractometer with a CCD detector, they inferred hügelite to be monoclinic, twinned by pseudomerohedry, with unit-cell parameters *a* = 31.066 (3) Å, *b* = 17.303 (2) Å, *c* = 7.043 (1) Å, β = 96.492 (2)°, *V* = 3761.6 (1) Å^3^, *Z* = 8 and *D*_calc_ = 5.74 Mg m^−3^. Thus, the unit-cell reported by them is twice as large as the original description. They reported that the crystal was twinned by pseudomerohedry, giving a very large pseudo-orthorhombic *C*-centred cell with the dimensions (transformed settings) *a* = 7.043 Å, *b* = 61.733 Å, *c* = 17.303 Å and γ = 90.02°. They further commented on and judged this twinning model, and presented a final refinement with a reasonable *R*_1_ value of 3.3%, nevertheless with a very low value of goodness-of-fit, *S* = 0.68 (thus, we assume the authors overestimated their data quality). We notice that Bruker *SHELXTL* Version 5 was used in the aforementioned study.

The new find of the rare mineral hügelite from the small uranium deposit Labská, Krkonoše Mts. (Czechia), prompted us to perform a new diffraction experiment, which revealed that the actual twinning in hügelite is different from the description presented by Locock & Burns (2003 ▸). Here we report on the results of our analysis that might help to understand the nature of the twinning in this mineral, as well as helping in future analyses of similarly twinned crystal structures.

## 2. Methodology   

### 2.1. Sample   

**Figure 1:**
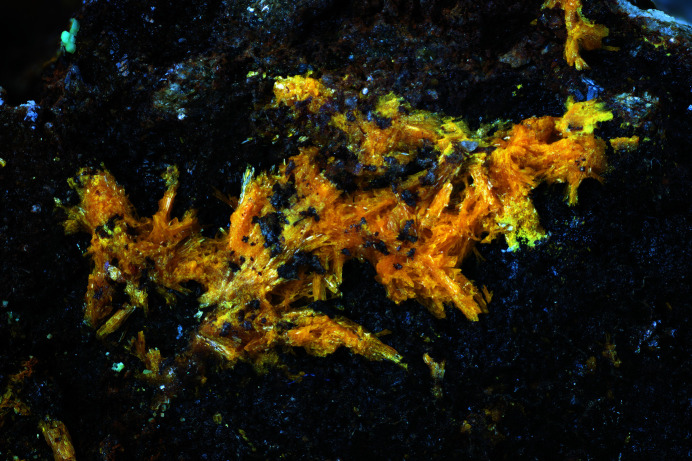
An aggregate of the tabular yellow crystals of hügelite from the Labská deposit. Field of view 7 mm (photo by P. Škácha).

The hügelite crystal used in this study was extracted from a specimen collected by Pavel Škácha from the Labská uranium deposit. This small uranium deposit is located approximately 5 km to the south of the town of Špindlerův Mlýn in the Krkonoše Mountains (Eastern Bohemia, Czechia). Hügelite forms elongated prismatic crystals apparently flattened on one of the prismatic faces. Crystals reach maximally up to 1 mm across (Fig. 1 ▸). They are dark orange in colour. Hügelite was found on a few specimens only, associated with more abundant dumontite. Phosphuranylite and saleéite were also identified in the association.

### 2.2. Single-crystal X-ray diffraction   

We studied two tiny crystals of hügelite from the Labská deposit. While the first crystal (hereafter denoted Labská I) of approximate dimensions 0.038 × 0.013 × 0.007 mm was later found to be affected by twinning, the second crystal (Labská II), having similar, but somewhat larger, dimensions (0.067 × 0.018 × 0.005 mm) was found later on to be untwinned. Diffraction experiments were carried out at room temperature with a Rigaku SuperNova single-crystal diffractometer. The diffraction experiment was carried out using Mo *K*α radiation (λ = 0.71073 Å) from a micro-focus X-ray tube collimated and monochromated by mirror optics and detected by an Atlas S2 CCD detector.

For both experiments, ω-rotational scans (of frame width 1°) were adopted and a full sphere of the diffraction data was collected. For the larger crystal, Labská II, an increased counting time per frame, equal to 840 s (compared to 400 s for Labská I), a high-sensitivity mode of the CCD detector (binning of pixels 2×2, with a high-gain option) and high-redundancy of the data set (∼7) were used to reveal even weak reflections.

The diffraction experiment for Labská I, as expected, due to previous results given by Locock & Burns (2003 ▸), provided a particularly complicated diffraction pattern, caused by twinning due to reticular merohedry (Petříček *et al.*, 2016 ▸). The studied crystal was found to be monoclinic, but with different unit-cell parameters than given by Locock & Burns (2003 ▸). Actually, the unit-cell parameters found are *a* = 7.0189 (7) Å, *b* = 17.1374 (10) Å, *c* = 8.1310 (10) Å and β = 108.904 (10)°, with *V* = 925.29 (15) Å^3^ and *Z* = 2 (Table 1 ▸). The reticular twin (diffraction type II) was found by the routine implemented in the *JANA2006* program (Petříček *et al.*, 2014 ▸). The twin operation is a mirror in the [100] direction; the second twin domain can be obtained by the matrix (−1 0 0 | 0 1 0 | 0.75 0 1), simulating a pseudo-orthorhombic supercell, which is eight times larger than a real (sub)cell (*a* = 7.019 Å, *b* = 17.137 Å, *c* = 61.539 Å and β = 90.02°). Therefore, during the data reduction, each twin domain was integrated alone and later imported into the *JANA2006* program utilizing the already known twin matrix that helped to define the orientation of each unit cell, thus resolving fully overlapped reflections. Those reflections, present in both data blocks, were included only one time to avoid their multiple occurrences in the refinement. The refinement in *JANA2006* including the twin model led to reasonable values of the twin fractions, *i.e.* 0.877 (1):0.123 (1) (Table 1 ▸); noticeably, the second twin domain is rather weak. The refinement, which took into account twinning, improved/smoothed slightly the difference Fourier; nevertheless, there are still false maxima due to poorly fitted absorption (apparent in the vicinity of the U atoms). The fact that intensities (and namely those of the contributing second domain) are quite weak resulted in the refinement converging to higher *R* = 7.58% for 2110 reflections with *I* > 3σ(*I*) (GOF = 1.63); noticeably, on first sight, we have also to take into account a different approach to the weighting scheme of the current refinement and a criterion for observed intensities.

The diffraction experiment for Labská II provided a similar unit cell, with *a* = 7.0258 (3) Å, *b* = 17.1769 (5) Å, *c* = 8.1463 (7) Å and β = 108.886 (5)°, with *V* = 930.18 (6) Å^3^ and *Z* = 2 (Table 1 ▸). The second experiment indicated that the crystal is less affected by the twinning than the first crystal; the current unit cell indexed 82% of all observed reflections compared to 29% indexed from the first experiment. The reciprocal space projections and reconstructions did not reveal any important contribution of the second domain; this was also proven later from the refinement. The second refinement in *JANA2006* converged to excellent agreement factors (Table 1 ▸). It is noteworthy that the GOF for the Labská II refinement is 1.07 for all 19 152 reflections.

Structure solution for the Labská I crystal was carried out using the intrinsic phasing algorithm of the *SHELXT* program (Sheldrick, 2015 ▸); refinement of Labská II was carried out using the model obtained for Labská I. Details of the data collection and refinement for both crystals are given in Table 1 ▸, final atomic coordinates and displacement parameters in Table 2 ▸, selected interatomic distances and hydrogen-bond parameters in Table 3 ▸, and a bond-valence analysis in Table 4 ▸. The bond-valence analysis was made following the procedure of Brown (2002 ▸, 2009 ▸) using bond-valence parameters pro­vided by Gagné & Hawthorne (2015 ▸). The formula of the crystal studied, based on refined occupancies and bond-valence calculations, is Pb_2_(UO_2_)_3_O_2_[(As_0.583_P_0.417_)O_4_)]_2_(H_2_O)_5_ (*Z* = 2 and *D*_calc_ = 5.669 Mg m^−3^).

**Figure 2:**
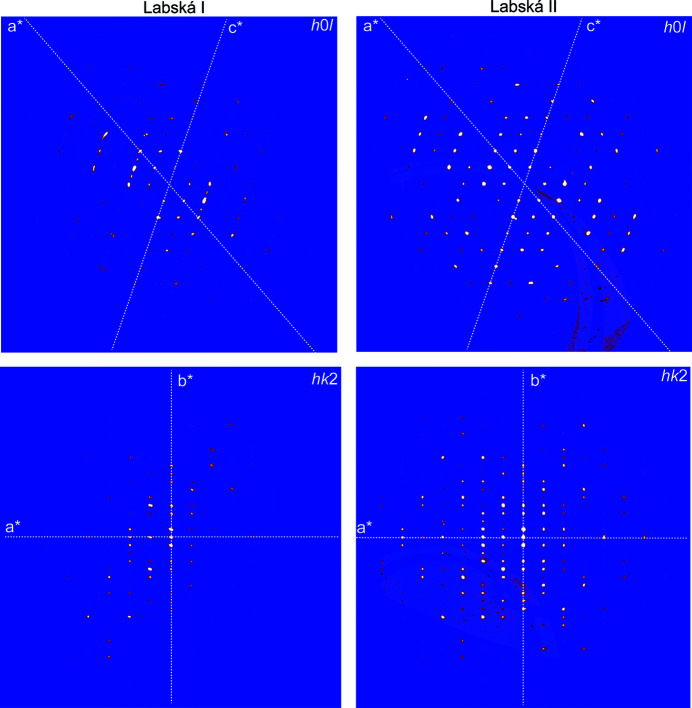
Twinning in hügelite, showing the reciprocal space reconstruction for the twinned (Labská I) and untwinned (Labská II) crystals. The twin contribution is easily visible in the case of the *h*0*l* layer. The biased intensities are apparent for the *hk*2 layer.

**Figure 3:**
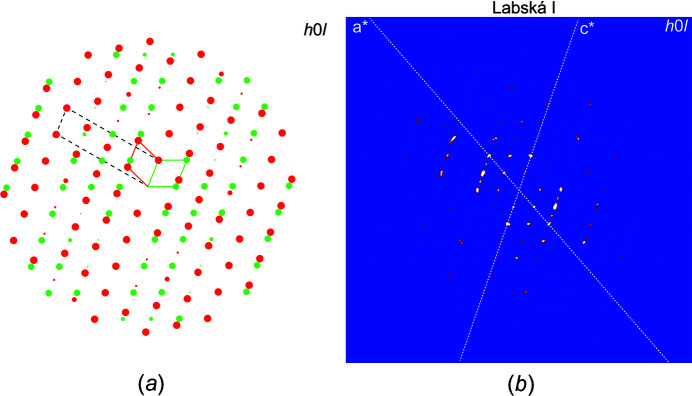
Twinning in hügelite, showing (*a*) the reciprocal space reconstructed from the intensity data in *JANA2006* displaying the contribution of the second domain (green). The correct unit-cell vectors are displayed as red and green rectangles. The choice for the centred cell of Locock & Burns (2003 ▸) is given in the black dashed lines. (*b*) The reciprocal space reconstruction of the corresponding *h*0*l* layer.

Twin contributions were evaluated visually using the reciprocal layers (Fig. 2 ▸) reconstructed from the CCD frames (*UNWARP* tool within the *CrysAlis* software; Rigaku OD, 2019 ▸) and by computer methods using the program *JANA2006* (Fig. 3 ▸). Twinning and the extra reflections due to twinning are easily visible for the Labská I crystal at the *h*0*l* and *hk*2 layers, for instance, while Labská II provided unbiased frames (Fig. 3 ▸). The presence of additional reflections can bias the indexing algorithms, because it simulates the larger unit-cell parameter. While the refinement of the Labská I crystal returned the refined twin fractions 0.877 (1) and 0.123 (1) (Table 1 ▸), the second crystal showed a negligible contribution of twinning only when a mirror operation was taken into account, Tw_vol1_/Tw_vol2_ = 0.9994 (5)/0.0006 (5).

## 3. Results   

**Figure 4:**
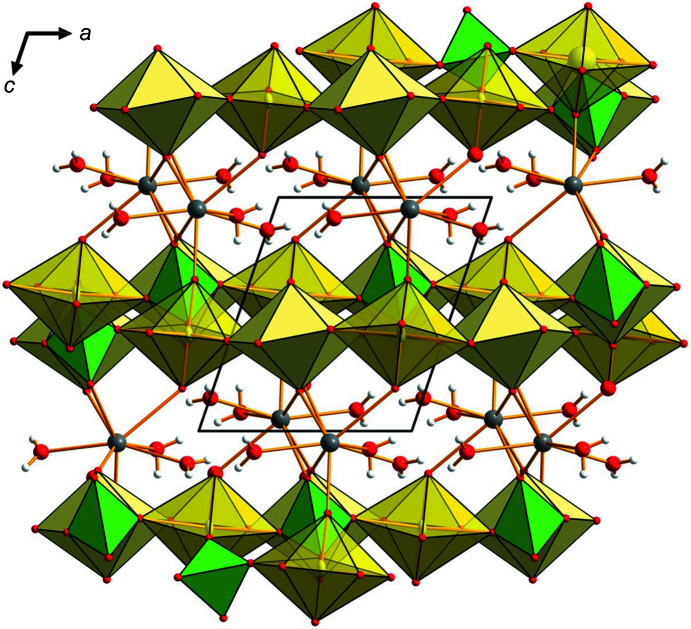
The crystal structure of hügelite projected down the monoclinic *b* axis. UO_7_ bipyramids are shown in transparent yellow, UO_8_ bipyramids are opaque yellow, (As/P)O_4_ tetrahedra are green, Pb atoms are dark gray (shown as displacement ellipsoids at the 75% probability level), H atoms are light gray and O atoms are red. The unit-cell edges are outlined with solid black lines. H⋯*A* bonds have been omitted for clarity.

Our description of the twinning in hügelite leaves the structure model proposed by Locock & Burns (2003 ▸) unchanged. The structure possesses uranyl–arsenate sheets with a phosphuranylite topology (Burns, 2005 ▸; Lussier *et al.*, 2016 ▸), with Pb^2+^ cations located in the interlayer space between the infinite sheets (Fig. 4 ▸).

### 3.1. Twinning in hügelite   

The twin operation, *i.e.* a mirror in [100], leads to a rather large supercell, with *V* ∼ 7400 Å^3^. There is a clear relationship between the unit cell derived by Locock & Burns (2003 ▸) and the supercell found in our study. The cell of Locock & Burns (2003 ▸) is half the volume of the supercell of our choice: our cell thus represents a real cell of hügelite, while the cell of Locock & Burns is a result of twinning (Fig. 3 ▸); the unit cell of Locock & Burns (2003 ▸) (*a* = 30.993 Å, *b* = 17.159 Å, *c* = 7.022 Å and β = 96.44), when applied a mirror in (001), leads to the same supercell as in the current study. The correct description of twinning in hügelite confirmed the originally reported unit cell (Walenta, 1979 ▸), having a unit-cell volume of ∼930 Å^3^. The correct unit cell of hügelite (*V* ∼ 930 Å^3^), when compared to dumontite (*V* ∼ 920 Å^3^), confirms that these two minerals are isotypic As- and P-dominant analogs, respectively. The increase of the unit-cell volume towards the As end member (hügelite), due to the larger effective ionic radius of As^5+^ compared to P^5+^, is apparent. It should be noted that the currently investigated crystal of hügelite is not an end member of the solid-solution series, based on the site-scattering refinement (Tables 1 ▸ and 2 ▸).

### 3.2. Hydrogen bonding in hügelite   

Although hügelite is a highly absorbing substance, even for Mo X-rays (μ = 46.30 mm^−1^), final difference-Fourier calculations revealed few maxima assignable to H atoms around those O atoms that belong, according to the bond-valence analysis, to H_2_O groups. Because it was impossible to freely refine all the parameters of the H atoms, they were refined using restrictions available in *JANA2006* for the hydrogen-bond geometry. Therefore, the scheme presented should be considered as an approximation at best. We also have to emphasize that the higher BV sums for both H atoms and the donor O atoms resulted from the used restraint on the *A*—H bond length taken as 0.82 Å as a conservative value for the hydrogen-bond length from X-ray analysis.

The hydrogen-bonding scheme can be deduced from the results of the bond-valence analysis (Table 4 ▸). There are three symmetrically independent H_2_O sites (corresponding to O5, O10 and O12), indicating five H_2_O molecules per unit cell for *Z* = 2. While atom O5 seems to be three-coordinated (one bond from Pb1 and two bonds to H1O5 and H2O5), atoms O10 and O12 are five- and four-coordinated, respectively. According to the terminology introduced by Schindler & Hawthorne (see, for example, Schindler & Hawthorne, 2008 ▸), O5 represents the transformer H_2_O group, while O10 represents an inverse transformer and O12 represents a nontransformer H_2_O group. Therefore, the current results are in contrast to the theoretical predictions made by Schindler & Hawthorne (2008 ▸), who concluded that hügelite should contain five inverse transformer (H_2_O) groups, based on the bond-valence approach. The above-mentioned scheme should be taken as a best-available model only. Due to underbonding of the O1 *U*_eq_ and O2 *U*_eq_ atoms (with corresponding low BV sums; Table 4 ▸), we can speculate about a somewhat different configuration, involving also the two O atoms *U*_eq_. Nevertheless, for such a task, employment of advanced theoretical approaches, as used recently for the uranyl phosphate mineral phurcalite (Plášil *et al.*, 2020 ▸), would be necessary.

## 4. Implications – processing the data using *JANA2006* to reveal the nature of twinning   

**Figure 5:**
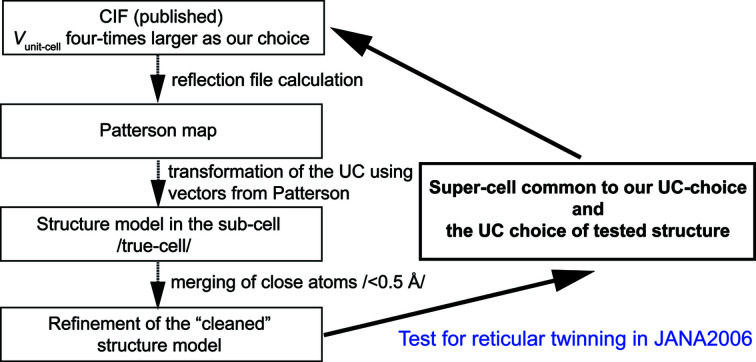
Diagram displaying the procedure for twin-testing in *JANA2006*.

Despite the fact that we did not have an original reflection file for the refinement of Locock & Burns (2003 ▸), the software we used for the structure analysis, *JANA2006*, enables us to perform a check for twinning in their structure, based on the available crystallographic information file (CIF). We have to emphasize here that the warning for the hidden translation symmetry in the CIF file of Locock & Burns (2003 ▸) was also indicated by *PLATON* ADDSYM (a quick test in IUCr *checkCIF*), returning the B Alert: ‘PLAT113_ALERT_2_B ADDSYM Suggests Possible Pseudo/New Space Group P2_1_/m Check Note: (Pseudo) Lattice Translation Implemented’. The entire procedure we followed in *JANA2006* is displayed in Fig. 5 ▸. The first step involves a calculation of the theoretical reflection file (Mo *K*α, full sphere) based on the atom positions in the CIF of Locock & Burns (2003 ▸). The next step involves the calculation of the Patterson map. As the autoconvolution of the electron density itself, it provides important information of the real metrics and can thus reveal the real periodicity features underlying the data. This analysis showed three pronounced Patterson maxima; from them, assuming the omnipresent inversion in the Patterson map, we obtained three translation vectors: (0, 0, 0), (−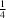, 0, 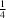), (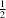, 0, 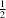), (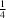, 0, −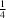). Those were used for the unit-cell transformation by the matrix | 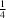 0 −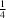 | 0 1 0 | 0 0 1 |. After axis transformation (*a*→*c*), we obtained the following cell: *a* = 7.043 Å, *b* = 17.30 Å, *c* = 8.1554 Å, α = 90°, β = 108.879° and γ = 90°. During the next step, the creation of the refinement reflection file (even if from the simulated data), there were 24 reflections found that violated the translation symmetry. Nevertheless, they were weak. The structure after the transformation into the smaller cell shows few atoms projected into very close positions (<0.5 Å). Merging the 24 atoms and refinement of the simulated structure led then to reasonably low *R* values (∼4.2% for 2771 reflections). The test for twinning by reticular merohedry/pseudomerohedry (Petříček *et al.*, 2016 ▸) readily revealed an orthorhombic supercell (7.043 Å, 17.302 Å, 61.733 Å, 90°, 89.98°, 90°), with a unit-cell volume eight times larger than the real cell. This supercell is a result of the twinning that can be described as a mirror in (100) of the *a* = 7.043 Å, *b* = 17.302 Å, *c* = 8.1554 Å, α = 90°, β = 108.879° and γ = 90° cell. Fig. 3 ▸(*a*) displays a pattern of the eight times larger cell; from this point of view, the cell choice of Locock & Burns (2003 ▸) is reasonable and is due to twinning, which had been present in their crystal without any shadow of a doubt.

## Supplementary Material

Crystal structure: contains datablock(s) global. DOI: 10.1107/S2052520621004091/ra5095sup1.cif

CIF file for the Labska I crystal (twinned). DOI: 10.1107/S2052520621004091/ra5095sup2.txt

Structure factors: contains datablock(s) I. DOI: 10.1107/S2052520621004091/ra5095Isup3.hkl

CCDC reference: 2078490

## Figures and Tables

**Table 1:** Details of the data collection and refinement for the two different crystals of hügelite from the Labská deposit

	Labská I	Labská II
Structural formula	Pb_2_(UO_2_)_3_O_2_[(As_0.597_P_0.403_)O_4_)]_2_(H_2_O)_5_	Pb_2_(UO_2_)_3_O_2_[(As_0.583_P_0.417_)O_4_)]_2_(H_2_O)_5_
*a*, *b*, *c* (Å), β (°)	7.0189 (7), 17.137 (1), 8.131 (1), 108.90 (1)	7.0258 (3), 17.1769 (5), 8.1463 (7), 108.886 (5)
*V* (Å^3^)	925.3 (2)	930.18 (10)
Space group	*P*2_1_/*m*	*P*2_1_/*m*
*Z*	2	2
*D*_calc_ (Mg m^−3^) for the above formula	5.667	5.669
Temperature (K)	297	297
Radiation type, wavelength (Å)	Mo *K*α, 0.71073	Mo *K*α, 0.71073
Crystal dimensions (µm)	38 × 18 × 7	67 × 18 × 5
Limiting θ angles (°)	2.90–29.48	2.38–29.78
Limiting Miller indices	–9 ≤ *h* ≤ 9, −23 ≤ *k* ≤ 23, −11 ≤ *l* ≤ 10	–9 ≤ *h* ≤ 9, −23 ≤ *k* ≤ 23, −11 ≤ *l* ≤ 10
No. of reflections measured	21 107	19 219
No. of unique reflections	4200	19 152
No. of observed reflections (criterion)	2107 [*I*_obs_ > 3σ(*I*)]	12 864 [*I*_obs_ > 3σ(*I*)]
Completeness, *R*_int_	0.93, 0.148	0.92, 0.069
Absorption correction (mm^−1^), *T*_min_, *T*_max_	46.59, 0.645, 1.000	46.30, 0.501, 1.000
*F*_000_	1319	1338
Parameters refined, restraints, constraints	134, 0, 13	141, 3, 27
*R*, *wR* (obs)	0.0763, 0.1837	0.0482, 0.1181
*R*, *wR* (all)	0.1591, 0.2263	0.0750, 0.1373
GOF obs, GOF all	1.67, 1.49	1.12, 1.07
Δρ_min_, Δρ_max_ (e Å^−3^)	−4.02, 6.61 (0.6 Å to U1)	−4.02, 5.04 (0.8 Å to U1)
Weighting scheme	*w* = 1/[σ^2^(*I*) + 0.0036*I* ^2^]	*w* = 1/[σ^2^(*I*) + 0.0036*I* ^2^]
Twin fractions 1, 2	0.8734 (12), 0.1266 (12)	
Twin matrix		
	\left({\matrix{ 1 & 0 & 0 \cr 0 & 1 & 0 \cr {0.75} & 0 & { - 1} \cr } } \right)	
		

**Table 2:** Atomic coordinates, isotropic and equivalent displacement parameters (Å) and site occupancies for the crystal structure of hügelite (Labská II)

Atom	*x*/*a*	*y*/*b*	*z*/*c*	*U*_iso_*/*U*_eq_
U1	0.20432 (12)	0.64347 (4)	0.41418 (11)	0.0148 (3)
U2	0.70073 (18)	0.75	0.40346 (17)	0.0179 (4)
Pb	0.63490 (15)	0.39415 (5)	0.05046 (13)	0.0277 (4)
As**	0.6916 (4)	0.55246 (16)	0.3866 (4)	0.0148 (11)
O1	1.008 (3)	0.75	0.380 (3)	0.016 (5)
O2	0.390 (3)	0.75	0.417 (3)	0.016 (5)
O3	0.807 (4)	0.75	0.632 (3)	0.032 (9)
O4	0.146 (3)	0.6333 (9)	0.184 (2)	0.022 (6)
O5	1.165 (4)	0.75	0.918 (3)	0.036 (10)
O6	0.590 (4)	0.75	0.170 (3)	0.025 (9)
O7	0.262 (3)	0.6491 (8)	0.646 (2)	0.028 (7)
O8	0.542 (3)	0.6162 (9)	0.435 (3)	0.033 (7)
O9	0.585 (3)	0.5111 (10)	0.203 (2)	0.035 (7)
O10	0.288 (4)	0.3429 (15)	0.088 (3)	0.067 (12)
O11	0.863 (3)	0.6154 (10)	0.374 (3)	0.037 (8)
O12	0.999 (3)	0.4474 (12)	0.137 (3)	0.044 (8)
O13	0.781 (2)	0.4928 (8)	0.543 (2)	0.021 (6)
H1o5	1.076 (9)	0.75	0.962 (10)	0.0315*
H2o5	1.123 (12)	0.75	0.8153 (17)	0.0315*
H1o10	0.2364	0.3051	0.03525	0.0778*
H2o10	0.2463	0.3656	0.1567	0.0778*
H1o12	1.0882	0.4218	0.1242	0.0534*
H2o12	1.0236	0.4910	0.1855	0.0534*

Note: (**) occupancy 0.597 (16) As/0.403 (16) P.

**Table:** 

U1—O4	1.808 (5)	U2—O1	2.238 (7)	Pb—O4^iv^	2.835 (6)
U1—O7	1.811 (6)	U2—O2	2.228 (6)	Pb—O5^v^	2.809 (3)
U1—O1^i^	2.245 (4)	U2—O3	1.796 (8)	Pb—O6^vi^	3.169 (4)
U1—O2	2.242 (4)	U2—O6	1.799 (7)	Pb—O7^ii^	2.455 (5)
U1—O8	2.387 (5)	U2—O8	2.611 (5)	Pb—O9	2.447 (6)
U1—O11^i^	2.366 (5)	U2—O8^iii^	2.611 (5)	Pb—O9^iv^	2.664 (5)
U1—O13^ii^	2.362 (5)	U2—O11	2.638 (5)	Pb—O10	2.670 (7)
〈U1—O_uranyl_〉	1.810	U2—O11^iii^	2.638 (5)	Pb—O12	2.585 (5)
〈U1—O_eq_〉	2.320	〈U2—O_uranyl_〉	1.798	〈Pb—O〉	2.704
		〈U2—O_eq_〉	2.494		
		As/P—O8	1.629 (6)		
		As/P—O9	1.617 (5)		
		As/P—O11	1.640 (5)		
		As/P—O13	1.589 (5)		
		〈As/P—O〉	1.619		

**Table:** 

*D*—H⋯*A*	*D*—H	H⋯*A*	*D*⋯*A*	*D*—H⋯*A*
O5—H1o5⋯O10^xv^	0.82 (2)	2.96 (6)	3.78 (1)	136 (4)
O5—H2o5⋯O3^v^	0.820 (16)	2.23 (6)	2.838 (9)	131 (7)
O10—H1o10⋯O6^vi^	0.82	2.55	2.973 (10)	113
O10—H2o10⋯O12^i^	0.82	2.21	2.876 (9)	138
O12—H1o12⋯O10^viii^	0.82	2.06	2.876 (9)	172
O12—H2o12⋯O13^vii^	0.82	2.22	2.746 (8)	122

Symmetry codes: (i) x-1, y, z; (ii) −*x* + 1, −*y* + 1, −*z* + 1; (iii) *x*, −*y* + 3 \over 2, *z*; (iv) −*x* + 1, −*y* + 1, −*z*; (v) −*x* + 2, *y* − 1 \over 2, −*z* + 1; (vi) -x+1, y-{\script{1\over 2}}, -z; (vii) -x+2, -y+1, -z+1; (viii) x+1, y, z; (xv) x+1, y, z+1.

**Table 4:** Bond-valence analysis (all values given in valence units, v.u.) for hügelite The bond-valence parameters are taken from Gagné & Hawthorne (2015 ▸).

	U1	U2	As	Pb	H1O5	H2O5	H1O10	H2O10	H1O12	H2O12	sum^–H^	sum^+H*^
O1	0.66	0.67									1.32	
O2	0.66	0.68									1.34	
O3		1.70				0.20					1.70	1.90
O4	1.65			0.16						0.20	1.82	2.02
O5				0.17	0.80	0.80					0.17	1.77
O6		1.69		0.08			0.20				1.76	1.96
O7	1.64			0.38							2.03	2.03
O8	0.49	0.30	1.27								2.06	2.06
O9			1.31	0.63							1.94	1.94
O10				0.24	0.20		0.80	0.80	0.20		0.24	2.04
O11	0.51	0.29	1.23								2.03	2.03
O12				0.29				0.20	0.80	0.80	0.29	2.09
O13	0.51		1.42								1.93	1.93
Sum	6.12	5.91	5.23	1.95	1.00	1.00	1.00	1.00	1.00	1.00		

Note: (*) idealized bond strengths taken from Brown (2002 ▸).
